# Effect of Recycling PET Fabric and Bottle Grade on *r*-PET Fiber Structure

**DOI:** 10.3390/polym15102330

**Published:** 2023-05-17

**Authors:** Nanjaporn Roungpaisan, Natee Srisawat, Nattadon Rungruangkitkrai, Nawarat Chartvivatpornchai, Jirachaya Boonyarit, Thorsak Kittikorn, Rungsima Chollakup

**Affiliations:** 1 Faculty of Engineering, Rajamangala University of Technology Thanyaburi, Pathum Thani 12110, Thailand; nanjaporn_r@rmutt.ac.th (N.R.); natee.s@en.rmutt.ac.th (N.S.); 2 Department of Textile Science, Faculty of Agro-Industry, Kasetsart University, Bangkok 10900, Thailandmill.nawarat@gmail.com (N.C.); 3 Kasetsart Agricultural and Agro-Industrial Product Improvement Institute (KAPI), Kasetsart University, Bangkok 10900, Thailand; aapjab@ku.ac.th; 4 Division of Physical Science, Faculty of Science, Prince of Songkla University, Songkhla 90110, Thailand; thorsak.k@psu.ac.th

**Keywords:** PET knitted fabric, PET bottle resin, compression, melt spinning, take-up speed

## Abstract

PET knitted fabric was melted and cooled by hot pressing at 250 °C to obtain a compacted sheet. Only white PET fabric (WF_PET) was used to study the recycling process by compression and grinding to powder and then melt spinning at different take-up speeds compared to PET bottle grade (BO_PET). PET knitted fabric had good fiber formability and was better suited for melt spinning of recycled PET (*r*-PET) fibers than the bottle grade. Thermal and mechanical properties of *r*-PET fibers improved in terms of crystallinity and tensile strength with increasing take-up speed (500 to 1500 m/min). Fading and color changes from the original fabric were relatively small compared with PET bottle grade. Results indicated that fiber structure and properties can be used as a guideline for improving and developing *r*-PET fibers from textile waste.

## 1. Introduction

Polyethylene terephthalate (PET) is mostly used in the textile and packaging industries. In 2019, production of PET was 30.5 million tons, with 60% used for fiber production in the textile industry and 30% used for the bottle industry, and this is expected to increase to 35.3 million tons by 2024 [1]. Asia Pacific has the largest PET packaging market share at 36.7%, followed by North America with 20.5% and Western Europe with 17.9% [2]. The PET-based packaging industry is expected to increase from 2020 to 2025 in the Middle East and Africa due to the growth of emerging socio-economies [2]. Recycled wastes are currently used to produce textiles instead of new materials, and PET has high resistance to atmospheric and biological agents. After disposal, PET fabrics slowly degrade in landfills with high environmental impact [3]. PET production is also an energy-intensive process that requires large amounts of crude oil [4]. Therefore, PET bottle recycling and reuse in the textile industry leads to a significant reduction in carbon emissions and raw material consumption [5,6].

In 2019, only 28.4% of recycled PET was made into sheets, fibers, films, and bottles, with the remainder discarded into the environment [7]. PET bottles are a ubiquitous post-consumer waste product [8] containing other polymer materials in the cap and label that can be easily removed. PET bottle wastes are not recycled for PET bottle production, with PET flakes generally utilized in the textile industry [9,10]. PET bottles are reused almost exclusively via primary or secondary recycling involving complex physical processes including sorting, cleaning, grinding into flakes, melting and reforming into filament, and then cutting into staple fiber for the textile or non-woven industries [11,12,13] to make secondary products such as carpet bottoms, sleeping bags and insulation materials, with recent application in the apparel industry. The associated energy consumption for these processes ranges from 8 to 55 MJ kg^−1^ [14]. Research articles concerning PET waste material derived products have focused on synthesizing other new polymeric materials in the fields of regenerative medicine and organic coatings or membranes [15,16].

PET fabric recycling requires separating other fiber materials such as cotton and PET blended fibers for Chief Value Cotton (CVC; with cotton making up over 50% of the blend) or Tetoron Cotton (TC; with PET making up over 50% of the blend) fabric types and also other synthetic fibers. Recycling of plain PET fabrics (100% PET called TK fabric) is performed through three methods as mechanical, chemical and thermo-mechanical [17]. The mechanical method offers the most simple and efficient approach including collection, sorting, cutting and shredding the fabric to fiber [18,19]. This shredded fiber is used for nonwoven textiles or insulation applications. The other part of this shredded fiber is opened in web form and fed into the spinning process to obtain coarse yarns. However, even when using clean pre-consumer PET waste, the mechanical recycling method still results in shorter fiber lengths, and recycled fibers are frequently blended with virgin fibers for apparel applications. The mechanical method is the simplest and most popular compared to the other two methods [20]. The cost is low but the economic value of the product is also low, requiring clean pre-consumer PET waste of the same color and fiber type. Chemical methods are widely used to recycle PET fiber production wastes including PET bottle wastes. Two common methods of depolymerization are glycolysis and methanolysis. Chemical recycling methods cause destructive mechanical, thermal, and electrical conductivity changes in PET properties [21]. Chemical method processes are complex and costly but the economic value of the final product is also high. Lastly, the thermo-mechanical method is based on remelting PET wastes to obtain re-granules that can be used in staple fiber production or in the plastic industry. Most thermo-mechanical methods are re-extrusion processes consisting of several steps of cutting, compacting/drying or drying and feeding to the extruder [17]. Thermo-mechanical methods are simpler than chemical methods and the economic value of the products is generally higher than obtained from mechanical methods. Therefore, they are most commonly used to recycle PET bottle wastes.

Disperse dyes are mostly used to dye PET fabrics. They are removed before fabric recycling otherwise the degree of polymerization (DP) may be reduced during the melting process [22]. The dye is dissolved and diffused into the PET fiber using heat or chemical substances that increase the amorphous areas for maximum dye penetration [23,24]. Polyester fiber is difficult to decolorize; therefore, removing dye from PET fabric waste requires reducing agents to elute the dye pigment. Part removal of dye in PET fabric gives light-colored remaining shades of yellow or gray. In this study, only white PET knitted fabrics (TK) were recycled using thermo-mechanical processes compared to the bottle pellet recycling process. The PET knitted fabric was adapted by compression and heating at melting temperature, then ground into particles and preheated under a hot air oven before melt spinning into filament to cause less degradation.

The recycling PET process generates molecular orientation and stress-induced crystallization that impact final mechanical properties of tenacity and dimensional stability [25]. The *r*-PET received from used bottles or cloth apparel can be categorized by (1) molecular weight, and (2) drawing ratio [26], with high molecular weight (M_w_) providing good tenacity during filament spinning. High M_w_ accompanied by high viscosity improves take-up speed and drawing ratio. The structure of PET fiber is mostly determined by the drawing process but the properties of spun fiber are also influenced by the spinning conditions that determine the maximum draw ratio and also modulus, strength, fiber diameter, and fineness [27]. The quality of the raw materials through sorting, collecting, and material preparation processes also influences fiber properties and operational costs. Thus, optimizing selective methodology in the material preparation step is important to minimize production costs.

Correlations among processing temperature, take-up speed, and fiber properties were investigated using differential scanning calorimetry (DSC) thermal analysis. Recycled filament properties are always reduced at each stage of thermal processing. This study investigated the appropriate processing parameters of melt spinning to compensate for the inevitable property losses of recycled filaments. Fiber characteristics of bottle PET grade were compared with recycled PET from fabrics. The parameters and fiber properties of recycled TK fabric (100% PET fabric) were also investigated.

## 2. Materials and Methods

### 2.1. Materials

White polyester knitted fabrics (code WF_PET) at basis weight 136.87 g/m^2^ were sourced from the stock of Jong Stit Co., Ltd., Bangkok, Thailand, while recycled bottle PET (BO_PET) was obtained from Teijin Co., Ltd., Bangkok, Thailand.

### 2.2. Preparation Parameters of r-PET Knitted Fabric Powder 

PET knitted fabric was softened by compaction at 230 °C for 3 min and then at 250 °C for 5 min before cooling to room temperature for 5 min to solidify the molten compressed sample, as shown in Table 1. The sheet of PET knitted fabric was then ground to powder and the thermal and melt flow properties of *r*-PET powder were characterized using a differential scanning calorimeter (DSC, Model 200F3, Netzsch™, Selb, Germany) with a scanning rate of 10 °C/min at 30–300 °C. A rheometer (C.B.N. Engineering Ltd., Bangkok, Thailand) was used to measure the melt flow index following ASTM D 1238.

### 2.3. Melt Spinning and Characterization of r-PET Fibers Prepared from r-PET Powder

Before preparing the fiber samples, the initial *r*-PET was preheated and dehumidified under a hot air oven at 80, 100, 120, and 140 °C for 1 h. Preheating and completion of the crystalline phase were necessary to prevent solid state formation during heating. The *r*-PET powder was prepared using a melt spinning machine (Thermo Haake Polydrive, Karlsruhe, Germany). The spinneret was set at a temperature of 270 °C and a throughput rate of 0.21 g/hole/min, with draw ratios of 500, 1000, and 1500 m/min (Table 2).

### 2.4. Characterization of r-PET Fibers Prepared from r-PET Powder 

The *r*-PET fibers prepared from *r*-PET powder of both WF_PET and BO_PET samples at different take-up speeds were characterized as below.

#### 2.4.1. Differential Scanning Calorimetry (DSC)

The melting, crystallization, and glass transition behavior of the *r*-PET filaments from different *r*-PET powders were studied using a differential scanning calorimeter (DSC, Model 200F3, Netzsch, Burlington, VT, USA). The specimen was heated in an aluminum pan from room temperature to 200 °C at a rate of 10 °C/min and then cooled to 0 °C. Heat history was recorded before reheating to 200 °C at the same rate. Cold crystallization and melting temperatures (T_c_ and T_m_, respectively) were obtained from the DSC curves. Delta H_m_, as the melting enthalpy (J/g), was used to calculate the percentage crystallinity, as shown in Equation (1):

(1) $$\%Xc=\frac{∆H_{m}}{{∆H_{m}}^{0}}\times 100\%$$

where ΔH_m_ [J/g] is the peak area (melting enthalpy) and ΔH_m_^0^ [J/g] is the melting enthalpy of a perfect PET crystal, equal to 140.1 J/g [27].

#### 2.4.2. Fiber Morphology under Optical Microscope

Cross section and longitudinal section fiber morphologies of all *r*-PET filaments of knitted fabric and bottle grade were studied after melt spinning under an optical microscope (Olympus Model GX41, Tokyo, Japan). Fiber diameter was measured using software and fiber fineness (denier) was calculated using Equation (2):

(2) $$\mathrm{Mass}=\mathrm{Denier}=\rho \;\times \;\frac{\pi a^{2}}{4}\;\times \;900,000$$

where denier is a direct measure of linear density as substance mass per unit volume (g/cm^3^), and a is the diameter (cm). In Equation (3):

(3) $$\rho =\frac{\mathrm{mass}}{\pi (\frac{a^{2}}{2})\times L}$$

where mass (g) is defined as the amount of matter in a substance, a is the diameter (cm), and L is the length (cm).

#### 2.4.3. Mechanical Properties

Tensile strength and percentage elongation at break of *r*-PET filaments were measured following the standard method of ASTM D3822 using a tensile tester (Instron Model 5560, Massachusetts, USA).

#### 2.4.4. Color Properties

Color properties, based on the CIELAB color system (L*, a*, b* and C* values) of *r*-PET filaments, were investigated using an X-RITE Ci60 handheld color spectrophotometer (Ci60-XRMNN, Illinois, USA). Total color differences (ΔE*) were measured and calculated using a portable colorimeter (Hunter Lab Miniscan EZ 4500L, Reston, VA, USA), as shown in Equation (4):

ΔE* = [(ΔL*)^2^ + (Δa*)^2^ + (Δb*)^2^]^0.5^ (4)

### 2.5. Statistical Analysis 

Data were subjected to one-way analysis of variance in a completely randomized design (CRD) for each PET material, with statistical significance set at *p* < 0.05 in the SPSS 17.0 for Windows software (SPSS Inc.; Chicago, IL, USA). Duncan’s new multiple range test was used to determine significant differences between treatments.

## 3. Results and Discussion

### 3.1. Characterization of r-PET Powder Prepared from White PET Fabric

**Figure 1:**
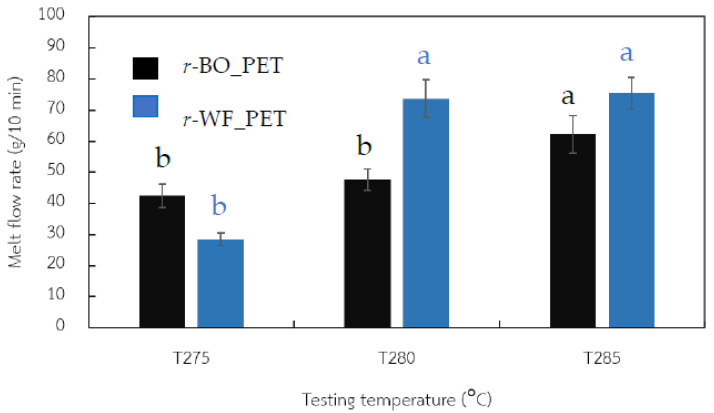
Melt flow index of *r*-PET powder prepared from white PET fabric compared to PET bottle grade as a reference, where different letters in the same PET indicate significant (*p* < 0.05).

Melt flow index (MFI) analysis was conducted under various temperatures to estimate the effect of processing temperature on *r*-PET fiber formation, as shown in Figure 1. Results revealed that the rheological values of bottle *r*-PET chips and fabric *r*-PET powder strongly depended on temperature. For every 5 °C adjustment (275 to 285 °C), results showed that the MFI value increased abruptly and was too high, especially at 285 °C. This suggested that the samples were sensitive and had limited residence time in a hot cylinder [28]. The overall MFI value for the *r*-PET powder sample prepared from fabric was higher than *r*-PET from bottles and depended on the original source. In melt spinning, the type of *r*-PET sample impacts the performance of the processability [29].

**Figure 2:**
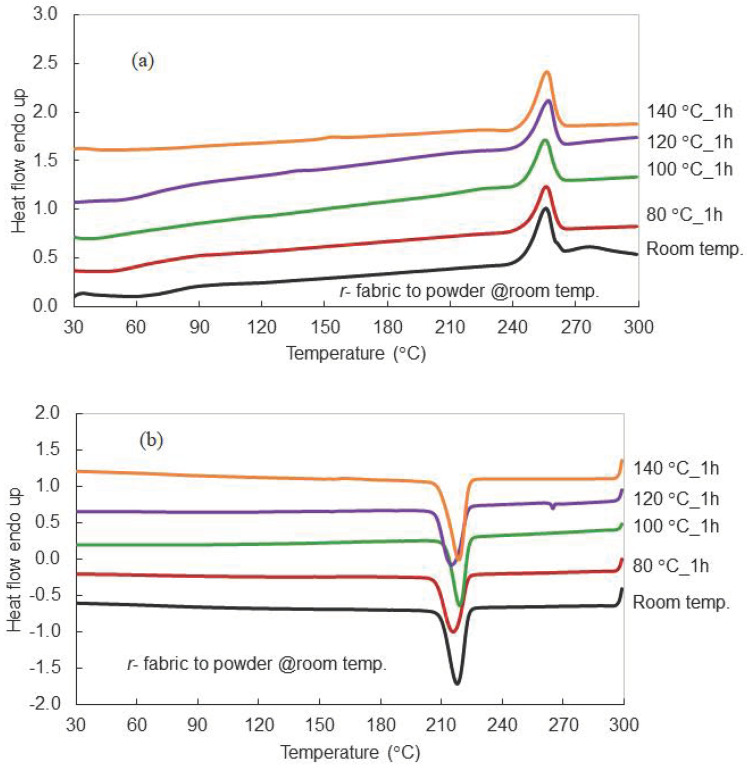
DSC thermograms of *r*-PET powder prepared from white PET fabric after recrystallization at different temperatures, (**a**) heating thermograms and (**b**) cooling thermograms.

**Figure 3:**
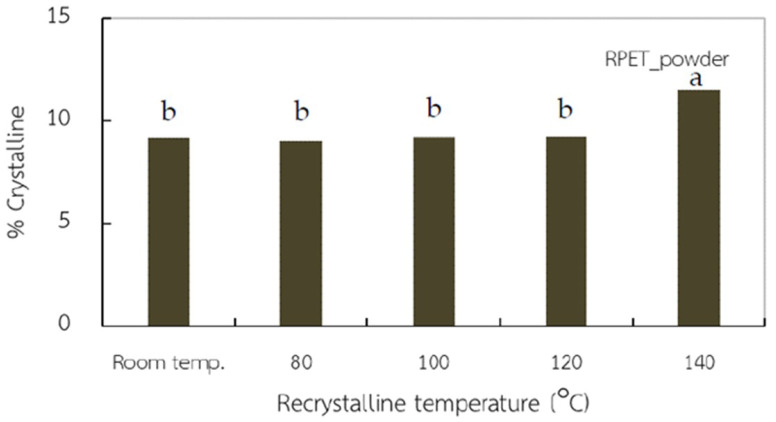
Crystalline percentages of *r*-PET powder prepared from white PET fabric after recrystallization at different temperatures, where different letters in the same property indicate significant (*p* < 0.05).

DSC measurements, shown in Figure 2 and Table 3, were used to study the effect of recrystallization temperature on the thermal behavior of *r*-PET powder made from compressed PET fabric. Generally, PET has a low crystallization rate [29], especially in the melting process. Therefore, it is necessary to preheat PET to clear the cold crystallization area before proceeding. Results showed that only melting and cooling temperatures appeared for all conditions. Surprisingly, cold crystallization did not form in these thermograms [30]. However, an in situ heating and cooling test of *r*-PET powder showed a higher order structure with excellent recrystallization at 140 °C, where molten polymer expressed the highest crystallinity content (Figure 3). PET materials have broad intrinsic thermal properties because the positions of T_g_, T_cc_, T_m_, and T_c_ are uncertain. Both kinetic and mechanism crystallization have different related amorphous/crystalline proportions, time-temperature-pressure, and environmental systems [31,32,33,34,35]. Previous research attempted to optimize crystallinity with thermal processing. Jabarin [36] reported that growth crystallization could be induced in the range 120–180 °C.

### 3.2. Effect of Melt Spinning of *r*-PET Fibers Prepared from White PET Fabrics

It is well known that *r*-PET fibers can be obtained from PET bottles [34]. Our research attempted to spin continuous filament from *r*-PET fabric powder. At the beginning of the process, the physical and thermal properties were different, affecting the spinnability. For example, the formation of a bottle needs proper flow behavior according to the blowing manner, similar to fiber production. In general, the MFI of spinning is higher than for the blowing method. Results in Table 4 show that *r*-PET fabric powder performed better than *r*-PET bottle grade when subjected to increasing winding speeds from 500 to 1500 m/min. The theory suggests that increasing elongational tension by stretching corresponds to molten flow behavior and solidification [37]. In this case, formation from compressed fabric powder occurred without any chemical modification compared to bottle grade.

**Figure 4:**
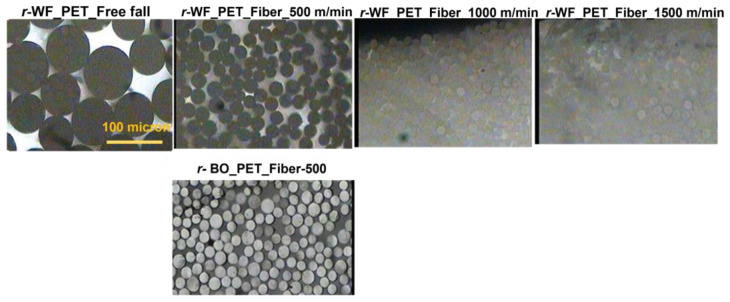
Cross sections of *r*-PET fibers prepared from white PET fabric using bottle grade as a reference at different take-up speeds under light microscope.

**Figure 5:**
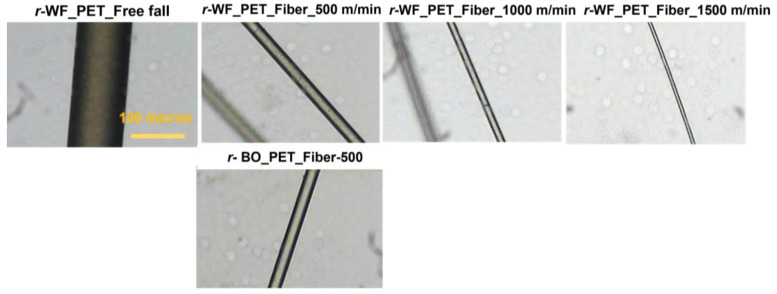
Longitudinal sections of *r*-PET fibers prepared from white PET fabric using bottle grade as a reference at different take-up speeds under a light microscope.

Captured pictures along cross section and longitudinal fibers are shown in Figure 4 and Figure 5, while fiber fineness results in Table 5 demonstrate the relationship between speed, diameter, and fiber evenness. Only *r*-PET fabric could be processed at 1500 m/min, while bottle *r*-PET had problems with discontinuous fiber formation caused by fiber breaking. An increment in stretching led to a decrease in fiber size, with the smallest size 18.97 denier (Table 5). However, the apparent morphology of *r*-PET fiber showed good uniformity and distribution.

**Figure 6:**
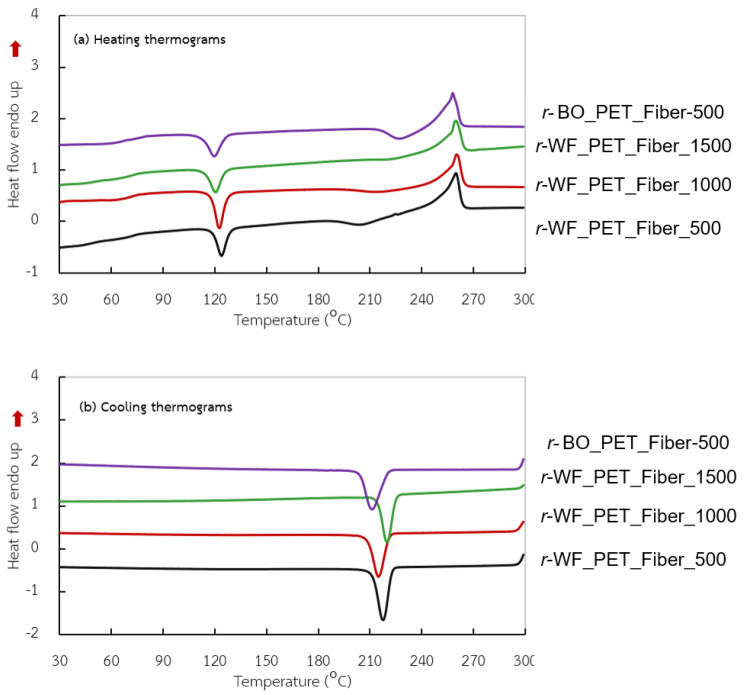
DSC thermograms of *r*-PET fibers prepared from white PET fabric using bottle grade as a reference at different take-up speeds.

**Figure 7:**
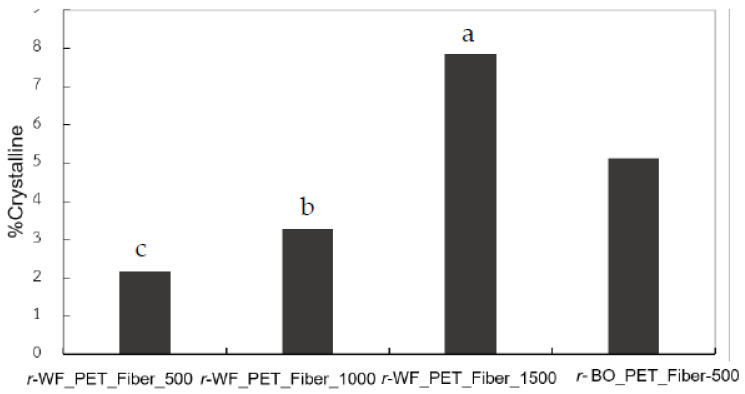
Crystalline percentages of *r*-PET fibers prepared from white PET fabric using bottle grade as a reference at different take-up speeds, where different letters in same property indicate significant (*p* < 0.05) difference.

The DSC thermograms of the r-PET as spun fiber at different take-up speeds are plotted in Figure 6, accompanying the thermal data in Table 6. In Figure 6a, two significant peaks appeared during heating up and one peak when cooling down at a constant rate of 10 °C/min. The thermal histories located cold crystallization (T_cc_) of the exothermic area at 122–123 °C in all samples, referred to as incomplete structures viz amorphous or imperfection. At 500 m/min, a large exothermic position took place during heating, while the melting temperature (T_m_) broadened exothermically and vice versa. At 1000 and 1500 m/min, the T_cc_ area of *r-* fabric PET tended to decrease. This outcome related to orientation and crystalline percentage, as illustrated in Figure 7. The melting temperature (T_m_) was constant for all samples, while the cooling temperature (T_c_) depended on the type of *r*-PET and stretching speed. Fibers produced from different fabrics had diverse thermal properties. Microstructures such as perfect crystal, crystallinity, orientation, and relative factors could be improved by enhancing processability, leading to higher order structure [34,35,38,39].

**Figure 8:**
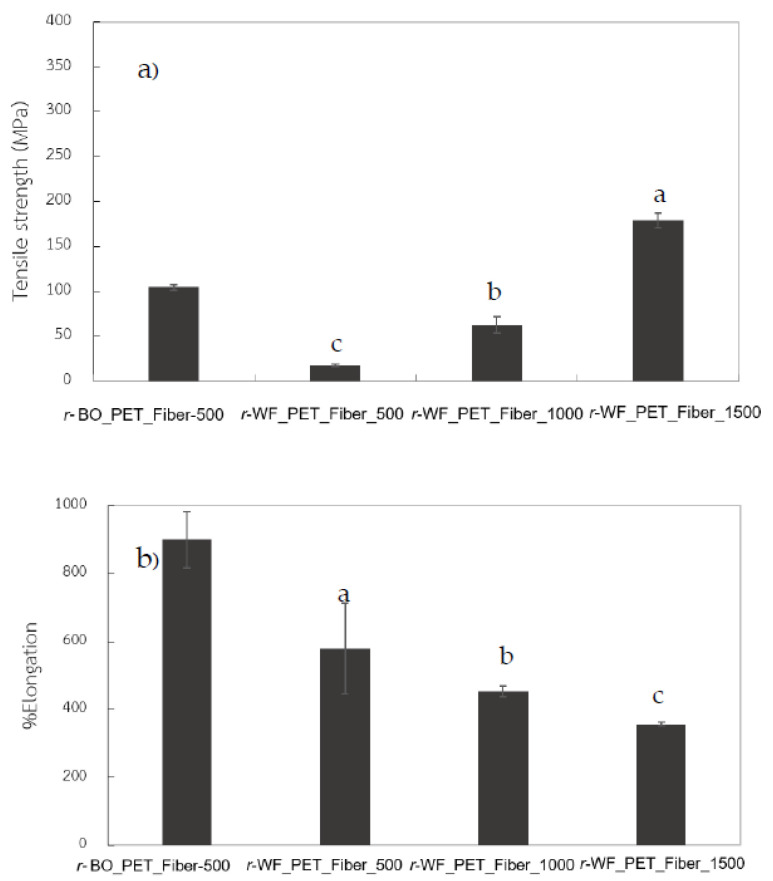
Tensile strength (**a**) and percentage elongation (**b**) of *r*-PET fibers prepared from white PET fabric and bottle reference at different take-up speeds, where different letters in same property indicate significant (*p* < 0.05) difference.

The mechanical properties of all *r*-PET samples were measured under tensile mode to assess the possibility of utilizing the fabric in textile products. Our novel method could be used to replace recycling PET from bottles. Tensile strength at the break value of stretched fibers improved by increasing take-up speeds in the case of *r*-WF_PET fiber (Figure 8). The performance of fiber made from bottle grade was higher at 500 m/min, while the *r*-PET fabric improved when take-up speed reached 1500 m/min. By contrast, the elongation value decreased. Intrinsic polymer properties such as melt flow rate and molecular weight were different. The melt spinning process results confirmed that the alternative of *r*-PET knitted fabric to *r*-PET powder enhanced overall fiber operation, as a possible new method to produce *r*-PET fiber for sustainable textile products. Under the same spinning conditions, structural developments in individual *r*-PET fiber types were dissimilar because of differences in intrinsic polymer properties such as melt flow rate, molecular weight, impurities and crystallinity [36,38,39].

Color properties of all *r*-PET fibers prepared from all materials are shown in Table 7. Recycling processes of melt compression and then melt spinning at high temperature caused PET degradation, resulting in color change, especially b* (+*b*: yellow and −*b*: blue color value). Color change (ΔE*) of WF_PET powder after compression and grinding was 7.63 after a two-step recycling process. After melt spinning, color change (ΔE*) increased at different take-up speeds of 500 to 1000 m/min and then decreased from 1000 to 1500 m/min. A take-up speed of 1500 m/min gave a stable fiber orientation which improved mechanical and crystalline properties of *r*-PET fibers prepared from white PET fabric. ΔE* more than doubled at different take-up speeds for melt spinning of *r*-WF_PET fibers compared to *r*-PET fibers at take-up speed of 500 m/min prepared from recycled bottle resin (BO_PET). The C* value of *r*-WF_PET fibers after melt spinning increased (0.73 to 2.01), while C* value of *r*-BO_PET fibers decreased (2.60 to 1.59) because fabric additives such as brightness agents improved grossness compared to bottle grade.

## 4. Conclusions

PET powder was prepared from WF_PET fabric via the hot-pressing process at 250 °C (onset of T_m_ PET) to minimize thermal and internal shear degradation. The WF_PET fabric was melted and then cooled to form a pressed plate ready for powder grinding. During this preparation method, the recycled fabric was subjected to less thermal processing than recycled pellets prepared by twin-screw extrusion (the general method of recycling PET bottles). Compared to recycled BO_PET, this method showed good fiber formability and was better suited for melt spinning of *r*-PET fibers from fabric than from bottle grade. The recycled WF_PET fibers had improved crystallinity and tensile strength, with increased take-up speeds (500 to 1500 m/min) compared to BO_PET. The melt spinning process confirmed that an alternative of recycled WF_PET to *r*-PET powder enhanced the overall fiber operation, as a possible new method to produce *r*-PET fiber for sustainable textile production. Fading and color changes from the WF_PET fabric were small compared with BO_PET grade. Therefore, *r*-PET fiber spinning at optimal conditions is essential. However, the preparation process of PET pellets for dyed fabric using disperse dyes requires further study, with or without the decolorization process, to investigate *r*-PET fiber structure after melt-spinning.

## Figures and Tables

**Table 1:** Preparation parameters of *r*-PET knitted fabric powder using the heat pressing method.

Parameters	Setting
Processing temperature (°C)	230, 250
Preheat time 1st (min)	3
Pressure of preheat (Pa)	100
Full pressure 2nd (min)	5
Cooling time (min)	5

**Table 2:** Melt spinning parameters of *r*-PET powder compared to *r*-PET filament.

Parameters	Setting
Orifice configuration	Round (0.32 mm)
Spinning temperature (°C)	260/270/280
Through rate (g/hole/min)	0.21
Take-up speed (m/min)	500/1000/1500
Pressure (Bar) and Toque (Nm)	4 and 27

**Table 3:** DSC data of *r*-PET powder prepared from white PET fabric after recrystallization at different temperatures.

Sample Name	T_m_ (°C)	ΔH_m_ (j/g)	T_c_ (°C)	ΔH_c_ (j/g)
Room temp.	276.1	25.24	218.3	−43.69
80 °C_1 h	255.7	26.61	216.1	−47.68
100 °C_1 h	255.3	26.66	219.3	−41.72
120 °C_1 h	256.9	27.75	215.5	−49.89
140 °C_1 h	256.0	34.01	219.0	−57.10

**Table 4:** Spinnability of *r*-PET fibers prepared from white PET fabric using PET bottle grade as a reference at different take-up speeds.

Samples	Take-Up Speed (m/min)		
	500	1000	1500
PET bottle reference	O	X	X
White PET fabric	O	O	Δ

O: Good spinnability; X: Fair spinnability; Δ: Poor spinnability.

**Table 5:** Fiber fineness of *r*-PET fibers prepared from white PET fabric using bottle grade as a reference at different take-up speeds.

Sample Name	Fiber Fineness (Denier)
*r*-WF-PET_Free fall	2635.18 ± 0.12 ^a^
*r*-WF-PET_Fiber-500	224.48 ± 0.12 ^b^
*r*-WF-PET_Fiber-1000	58.99 ± 0.03 ^c^
*r*-WF-PET_Fiber-1500	18.97 ± 0.06 ^d^
*r*-BO-PET_Fiber-500	65.87 ± 0.07

Mean ± SD in the same white PET fabric superscripted with different letters are significantly (*p* < 0.05) different.

**Table 6:** DSC data of *r*-PET fibers prepared from white PET fabric using bottle as a reference at different take-up speeds.

Sample Name	T_cc_ (°C)	ΔH_cc_ (j/g)	T_m_ (°C)	ΔH_m_ (j/g)	T_c_ (°C)	ΔH_c_ (j/g)
*r*-WF-PET_Fiber_500	125.1	−34.06	259.9	37.10	215.1	−45.01
*r*-WF-PET_Fiber_1000	124.0	−39.44	260.1	44.01	218.2	−45.70
*r*-WF-PET_Fiber_1500	122.5	−35.52	259.6	46.50	220.1	−54.10
*r*-BO-PET_Fiber_500	120.0	−35.03	257.1	42.20	210.1	−46.20

**Table 7:** Color properties of *r*-PET samples prepared from white PET fabric using bottle grade as a reference at different take-up speeds.

Sample Name	L*	a*	b*	ΔE*	C*
WF_PET	87.66	1.88	−3.06		3.59
*r*-WF_PET_Compressed	91.52	0.13	−0.86	4.78	0.87
*r*-WF_PET_Powder	94.57	0.73	−0.03	7.63	0.73
	94.57	0.73	−0.03		0.73
*r*-WF_PET_Fiber_500	89.82	1.27	−0.18	4.78	1.28
*r*-WF_PET_Fiber_1000	86.23	1.47	0.62	8.40	1.59
*r*-WF_PET_Fiber_1500	87.35	0.39	1.98	7.50	2.01
*r*-BO_PET_Pellet	72.64	−2.44	0.89		2.60
*r*-BO_PET_Fiber_500	87.42	1.53	−0.45	15.36	1.59

## Data Availability

Data are contained within the article.
